# A Mixture of Topical Forms of Polydeoxyribonucleotide, Vitamin C, and Niacinamide Attenuated Skin Pigmentation and Increased Skin Elasticity by Modulating Nuclear Factor Erythroid 2-like 2

**DOI:** 10.3390/molecules27041276

**Published:** 2022-02-14

**Authors:** Hyoung Moon Kim, Kyung-A Byun, Seyeon Oh, Jin Young Yang, Hyun Jun Park, Moon Suk Chung, Kuk Hui Son, Kyunghee Byun

**Affiliations:** 1 Department of Anatomy & Cell Biology, College of Medicine, Gachon University, Incheon 21936, Korea; drmac12@me.com (H.M.K.); kabyun95@gmail.com (K.-A.B.); 2 Functional Cellular Networks Laboratory, Graduate School and Lee Gil Ya Cancer and Diabetes Institute, College of Medicine, Gachon University, Incheon 21999, Korea; seyeon8965@gmail.com (S.O.); roswellgirl111@gmail.com (J.Y.Y.); 3 Maylin Anti-Aging Center Apgujeong, Seoul 06005, Korea; parmani@naver.com; 4 I’ll Global Co., Inc., Seoul 06532, Korea; sugi0821@empas.com; 5 Department of Thoracic and Cardiovascular Surgery, Gil Medical Center, Gachon University, Incheon 21565, Korea

**Keywords:** niacinamide, nuclear factor erythroid-2-related factor 2, polydeoxyribonucleotide, skin pigmentation, skin pigmentation, vitamin C

## Abstract

It is well-known that increased oxidative stress caused by ultraviolet B (UV-B) radiation induces melanogenesis and activates metalloproteinases (MMPs), which degrade collagen and elastin fibers, leading to decreased skin elasticity. Various antioxidant agents, such as vitamin C and niacinamide, have been evaluated for use as treatments for photoaging or skin pigmentation. In this study, we evaluated the ability of a topical liquid formula of polydeoxyribonucleotide (PDRN), vitamin C, and niacinamide (PVN) delivered via a microneedling therapy system (MTS) to attenuate photoaging and pigmentation by increasing nuclear factor erythroid 2-like 2 (NRF2)/heme oxygenase-1 (HO-1) and decreasing MMP expression in a UV-B-radiated animal model. The effects of the PVN were compared with those of individual PDRN and hydroquinone (HQ) compounds. The expression of NRF2/HO-1 significantly increased in response to HQ, PDRN, and PVN in UV-B-radiated animal skin. The activity of nicotinamide adenine dinucleotide phosphate hydrogen oxidase decreased in response to HQ, PDRN, and PVN, and the superoxide dismutase activity increased. The expression of tumor protein p53 and microphthalmia-associated transcription factor and tyrosinase activity decreased in response to HQ, PDRN, and PVN, and this decrease was accompanied by decreased melanin content in the skin. The expression of nuclear factor kappa-light-chain enhancer of activated B cells and MMP2/3/9 decreased in response to HQ, PDRN, and PVN in UV-B-radiated skin. However, the expression of collagen type I α1 chain and the amount of collagen fibers that were evaluated by Masson’s trichrome staining increased in response to HQ, PDRN, and PVN. The contents of elastin fibers, fibrillin 1/2 and fibulin 5 increased in response to HQ, PDRN, and PVN. In conclusion, PVN delivered via MTS led to decreased melanogenesis and destruction of collagen and elastin fibers by MMPs, and, thus, PVN decreased skin pigmentation and increased skin elasticity.

## 1. Introduction

Ultraviolet (UV) light leads to skin aging by increasing the production of reactive oxygen species (ROS), which upregulate the activity of collagenase and elastase [1,2]. Metalloproteinases (MMPs) and collagenases lead to the destruction of extracellular matrix (ECM) proteins and result in skin wrinkles. These enzymes also decrease elasticity in the skin, since collagen and elastin in the dermis determine skin elasticity [1,2,3].

The macroscopic characteristics of skin photoaging include wrinkle formation, rough texture, pigmentation, and loss of skin elasticity. Histological and ultrastructural studies have shown epidermal hyperplasia, damage, disorder of collagen fibers, and a large accumulation of abnormal elastic substances in connective tissue in photoaged skin [4,5]. However, these effects are less pronounced in the epidermis, owing to high turnover. In contrast, the dermal region is more susceptible to photodamage, which results in loss of skin resilience [6].

The increased activity of nuclear factor-κB (NF-κB) by ROS is one of the main signaling pathways that leads to the upregulation of MMPs in dermal fibroblasts [7].

Increased production of ROS by UV light also leads to enhanced melanogenesis [8]. ROS induced by UV radiation leads to increased activation of the tumor suppressor protein p53 [9]. The p53 upregulates proopiomelanocortin (POMC) and leads to increased cleavage of POMC into adrenocorticotropic hormone (ACTH) and α-, β-, and γ-melanocyte-stimulating hormones (MSHs) [10]. Increased α-MSH by UV radiation leads to enhanced activity of the melanocortin 1 receptor, which subsequently promotes the expression of microphthalmia-associated transcription factors (MITFs) [11,12]. MITFs, the main controllers of melanogenesis-related proteins, including tyrosinase and tyrosinase-related protein (TRP) 1/2, trigger melanogenesis [11,12].

Nuclear factor erythroid 2-like 2 (NRF2) is one of the main defensive systems that acts against ROS [13]. As a defense response to oxidative stress, NRF2 is activated by the release of Kelch-like ECH-associated protein (Keap), which activates NRF2 translocation to the nucleus, leading to the upregulation of various antioxidative enzymes and proteins [13]. NRF2 is involved in increasing levels of superoxide dismutase (SOD) (one of the primary antioxidant enzymes) and heme oxygenase-1 (HO-1) [14]. Moreover, it is well-known that increased HO-1 inhibits the activity of nicotinamide adenine dinucleotide phosphate hydrogen (NADPH) oxidase, which subsequently decreases the production of superoxide [15]. UV-B radiation leads to decreased expression of NRF2 in melanocytes [16,17].

Photoaging and skin pigmentation induced by UV light cause cosmetic problems; thus, many studies aiming to decrease photoaging with various agents or compounds have been performed.

Hydroquinone (HQ) has been used as a standard treatment for skin hyperpigmentation, since this compound leads to competitive inhibition of tyrosinase, which decreases melanin synthesis [18]. However, the chronic use of HQ induces irritant dermatitis [19,20]. Moreover, HQ induces permanent damage to melanosomes and leads to exogenous ochronosis [21]. Several reports have shown that HQ might be related to damage to renal and liver cells [22,23,24]. Moreover, there are concerns related to possible carcinogenicity induced by HQ [25]. Thus, the usage of HQ as a cosmetic additive is prohibited in various places, such as Australia, the UK, Europe, Asia, and Africa [26,27].

Polydeoxyribonucleotide (PDRN) is a combination of deoxyribonucleotides isolated from the testis of salmon [28]. PDRN is isolated from sperm DNA and purified at a high temperature, which guarantees that more than 95% of pure active substances are recovered, as well as inactivation of other proteins and peptides that could possibly induce immunological side effects [29]. The inactivation of proteins and peptides is associated with the safety of the PDRN. Moreover, spermatozoa are known to be the most suitable cells from which to source highly purified DNA while minimizing the risk of impurity [29]. PDRN is degraded to oligo- and mononucleotides by unspecific plasma DNA nucleases or nucleases in the cell membranes. By those degradation process, PDRN could have biological effects that act on adenosine A2A receptors [29]. PDRN has been reported to exhibit various effects, such as anti-inflammatory, angiogenesis promotion, anti-apoptotic, and tissue repair [30,31,32,33]. PDRN enhances the synthesis of nucleic acid as a source of pyrimidines and purines [34].

It has been reported that PDRN enhances the metabolism of fibroblasts and increases the synthesis of dermal matrix components [35,36,37]. PDRN inhibits the activity of elastase and MMP1, which increase skin elasticity and decrease wrinkling [38,39]. Moreover, PDRN reduces melanogenesis by decreasing the expression of MITF and the activity of tyrosinase, TRP1, and TRP2 [40].

Vitamin C leads to decreased skin wrinkling by increasing the proliferation and migration of dermal fibroblasts and enhancing the production of collagen and elastin [41,42]. Vitamin C leads to decreased melanin synthesis by inhibiting tyrosinase0 [43].

Niacinamide (nicotinamide), which is mainly used as a vitamin B3 supplement, has been reported to have various cosmetic effects [44,45,46,47,48,49,50]. Niacinamide exhibits a protective effect against UV-light-induced DNA damage in epidermal melanocytes by increasing the components of various signaling pathways, such as sirtuin 1 (SIRT1) and NRF2 [44]. Niacinamide also decreased age-induced ECM changes by increasing collagen (types I, III, and V), elastin, and fibrillin (1 and 2) [45,46]. However, it has been shown to decrease the expression and activity of elastase and MMP (1, 3, and 9) in UV-A-radiated skin fibroblasts [45,46]. Moreover, niacinamide leads to skin whitening. Although consistent results related to decreasing tyrosinase activity have not been shown [47,48], melanin pigmentation is decreased by reducing the transfer of melanosomes from melanocytes to keratinocytes [49,50].

To increase the effect of individual compounds, such as PDRN, vitamin C, and niacinamide, on photoaging, the mixture of those compounds as topical formulations might be helpful to expect synergistic effects. We evaluated the effects of topical liquid forms of PDRN, vitamin C, and niacinamide (PVN) on attenuating photoaging and pigmentation in a UV-B-radiated animal model. We also compared the effect of the PVN with HQ and an individual PDRN compound. To deliver PVN more effectively, we also used a microneedling therapy system (MTS).

## 2. Results

### 2.1. PVN Showed Highest Effect on Decreasing Melanin Amount among Various Combinations of Niacinamide, Vitamin C, and PDRN

To find the most effective combination of niacinamide, vitamin C, and PDRN, we evaluated the amount of melanin in an α-MSH-treated human melanocyte (Supplementary Figure S1). First, we evaluated the melanin amount when a single compound (niacinamide, vitamin C, or PDRN) was used to treat human melanocytes. Melanin was significantly increased when treated with α-MSH, while it was decreased by niacinamide, vitamin C, and PDRN. The most prominent decreasing effect on melanin levels was shown when PDRN was used (Supplementary Figure S1A). Next, we evaluated the decrease in melanin caused by using combinations of two compounds: vitamin C+niacinamide, PDRN+niacinamide, and vitamin C+PDRN. The most significant decreasing effect was shown by vitamin C+PDRN (Supplementary Figure S1B). Thus, we compared the decreasing effect on melanin between vitamin C+PDRN and PDRN+vitamin C+niacinamide (PVN). PVN showed a stronger decreasing effect on melanin than vitamin C+PDRN (Supplementary Figure S1C). Since PDRN showed the most prominent effect of decreasing melanin as a single compound, we decided to compare the effect of PVN on decreasing melanin synthesis with single-compound PDRN and HQ.

### 2.2. PVN Increased the Expression of pNRF2 and HO-1 and Decreased NADPH Oxidase Levels in Both In Vitro and In Vivo Models of UV-B Radiation

**Figure 1:**
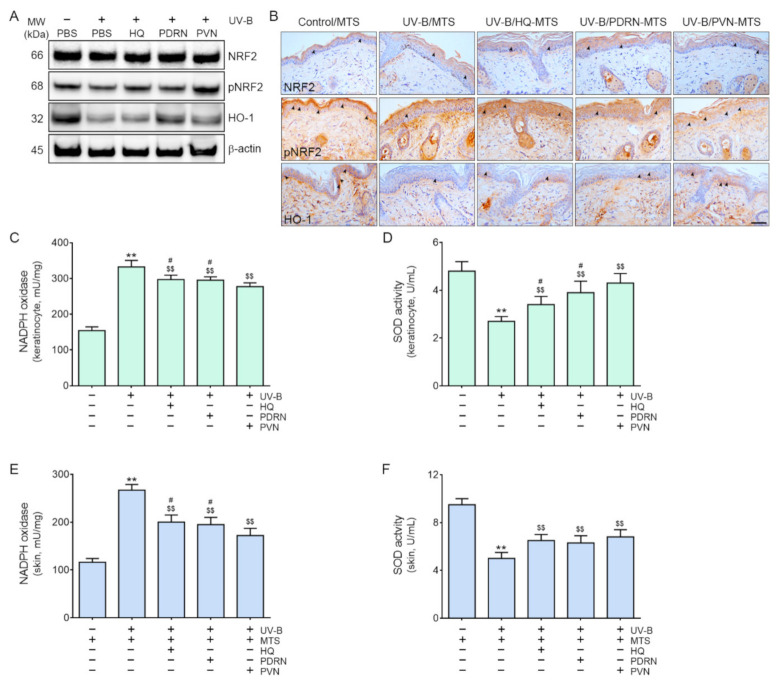
Regulation of the expression of NRF2 and HO-1, NADPH oxidase activity, and SOD activity by HQ, PDRN, and PVN treatment in UV-B-radiated human keratinocytes and animal skin. (**A**) Immunoblot analysis of NRF2, pNRF2, and HO-1 in human keratinocytes. (**B**) NRF2, pNRF2, and HO-1 expression in the epidermis of UV-B-radiated mice was determined by immunohistochemistry (scale bar = 100 µm). The arrows indicated positive cells of NRF2, pNRF2, and HO-1. (**C**,**D**) NADPH oxidase activity (**C**) and SOD activity (**D**) were determined in the UV-B-radiated human keratinocytes. (**E**,**F**) NADPH oxidase activity (**E**) SOD activity (**F**) were measured in the UV-B-radiated animal skin. Data are presented as the mean ± standard deviation; **, *p* < 0.01 vs. first bar; $$, *p* < 0.01 vs. second bar; #, *p* < 0.05 vs. 5th bar (Mann–Whitney U test). HO-1, heme oxygenase-1; HQ, hydroquinone; MTS, microneedling therapy system; MW, molecular weight; NADPH, nicotinamide-adenine-dinucleotide phosphate hydrogen; NRF2, nuclear factor erythroid-2-related factor 2; PBS, phosphate-buffered saline; PDRN, polydeoxyribonucleotide; pNRF2, phosphorylated nuclear factor erythroid-2-related factor 2; PVN, polydeoxyribonucleotide+vitamin C+niacinamide; SOD, superoxide dismutase; UV-B, ultraviolet B.

First, we evaluated whether UV-B radiation decreased NRF2 and HO-1 in primary normal human epidermal keratinocytes (Supplementary Figure S2A). After five minutes of UV-B radiation of the keratinocytes, they were treated with phosphate-buffered saline (PBS), HQ, PDRN, or PVN. The expression ratio of pNRF2 and NRF2 (pNRF2/NRF2) that was evaluated by immunoblotting assay significantly decreased in response to UV-B radiation and significantly increased in response to HQ, PDRN, and PVN (Figure 1A and Supplementary Figure S3A). The increasing effect of PVN was significantly higher than that of PDRN or HQ. The expression of HO-1 significantly decreased in response to UV-B radiation and significantly increased in response to the administration of HQ, PDRN, and PVN (Figure 1A and Supplementary Figure S3B). The increasing effect of the PVN was significantly higher than that of HQ.

Next, we evaluated whether NRF2 and HO-1 increased in the UV-B-radiated animal skin and decreased after PVN treatment (Figure 1B and Supplementary Figure S3C,D). To increase the penetration of PVN into the skin, we used a MTS. Moreover, we also delivered distilled water via MTS in the control or UV-B-radiated animal groups to minimize the bias which possibly caused by using MTS only in the experimental groups (HQ, PDRN, and PVN treated groups). The expression of pNRF2/NRF2 in the epidermis of UV-B-radiated animals was significantly lower than that of control animals (Figure 1B and Supplementary Figure S3C). The same expression significantly increased in response to the topical application of HQ, PDRN, and PVN. The increasing effect of the PVN was significantly higher than those of PDRN or HQ.

The expression of HO-1 in the epidermis of UV-B-radiated animals was significantly lower than that of the control animals, but the expression significantly increased in response to the topical application of HQ, PDRN, or PVN. The increasing effect of the PVN was significantly higher than that of PDRN or HQ (Figure 1B and Supplementary Figure S3D).

The activity of NADPH oxidase significantly increased in response to UV-B and was significantly decreased in keratinocytes by the administration of HQ, PDRN, or PVN (Figure 1C). The most prominent decreasing effect was shown in PVN-treated keratinocytes. The activity of SOD significantly decreased in response to UV-B and significantly increased in response to the administration of HQ, PDRN, and PVN (Figure 1D). The most prominent increasing effect was shown in PVN-treated keratinocytes.

The NAPDH oxidase activity in the skin of UV-B-radiated animals was significantly higher than that of normal control animals but significantly decreased in response to topical application of HQ, PDRN, and PVN. The most prominent decreasing effect was shown for PVN (Figure 1E). The activity of SOD in the skin of UV-B-radiated animals was significantly lower than that of normal control animals but significantly increased in response to topical application of HQ, PDRN, and PVN (Figure 1F). However, the increasing effects among HQ, PDRN, and PVN were not significantly different.

### 2.3. PVN Decreased Melanogenesis by Decreasing p53/MITF/Tyrosinase Expression in Both In Vitro and In Vivo Models of UV-B Radiation

**Figure 2:**
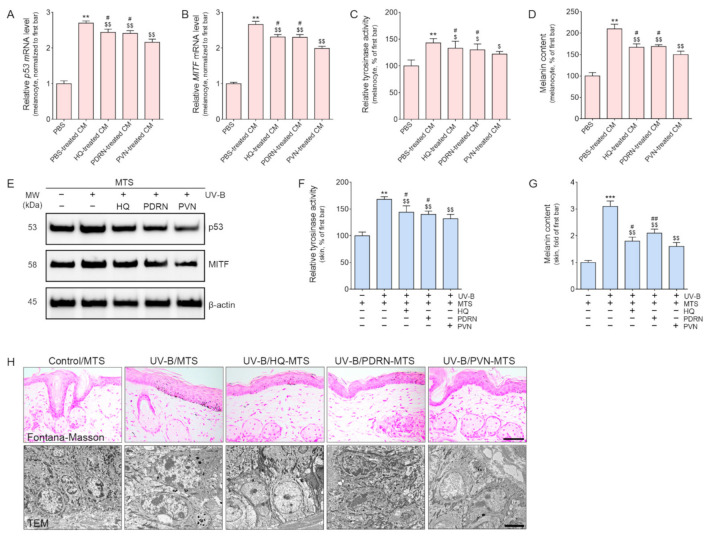
Regulation of the expression of p53 and MITF and tyrosinase activity by HQ, PDRN, and PVN treatment. (**A**,**B**) The mRNA expression levels of p53 (**A**) and MITF (**B**) were determined in the melanocytes treated with PBS or CM from UV-B-radiated keratinocytes. CM was obtained from a UV-B-radiated keratinocyte culture treated with HQ, PDRN, or PVN. The mRNA levels were normalized by Actb and expressed relative to the levels in the PBS group. (**C**,**D**) The tyrosinase activity (**C**) and melanin contents (**D**) determined in the melanocytes treated with PBS or CM from UV-B-radiated keratinocytes. (**E**) Immunoblot analysis of p53 and MITF determined in the UV-B-radiated animal skin. (**F**) The tyrosinase activity was evaluated in the UV-B-radiated animal skin. (**G**) The melanin contents evaluated with Fontana–Masson staining in the UV-B-radiated animal skin was exhibited as a graph. (**H**) The melanin content was assessed by Fontana–Masson staining (upper row; scale bar = 100 µm) and TEM (lower row; scale bar = 5000 nm). Melanin is stained as blackish dots in the Fontana–Masson staining. Data are presented as the mean ± standard deviation: **, *p* < 0.01 and ***, *p* < 0.001 vs. first bar; $, *p* < 0.05 and $$, *p* < 0.01 vs. second bar; #, *p* < 0.05 and ##, *p* < 0.01 vs^.^ 5th bar (Mann–Whitney U test). CM, conditioned medium; HQ, hydroquinone; MITF, microphthalmia-associated transcription factor; MTS, microneedling therapy system; MW, molecular weight; PBS, phosphate-buffered saline; PDRN, polydeoxyribonucleotide; PVN, polydeoxyribonucleotide + vitamin C + niacinamide; p53, tumor protein p53; TEM, transmission electron microscopy; UV-B, ultraviolet B.

PVN was applied topically. Thus, we assumed that keratinocytes in the epidermis might first be affected by the PVN and then affected by the keratinocytes afterward to secrete some effective factors that lead to decreased melanin synthesis in melanocytes. Thus, HQ, PDRN, or PVN was added to UV-B-radiated keratinocytes and then the supernatant from those cell cultures was collected after 24 h (Supplementary Figure S2B). The melanocytes were treated with the conditioned medium (CM) from keratinocyte culture or PBS. The expression of p53 and MITF, tyrosinase activity, and melanin contents was evaluated in melanocytes (Figure 2A–D). The mRNA expression of p53 and MITF was significantly increased in melanocytes treated with the CM from UV-B-radiated keratinocytes. These parameters significantly decreased in response to the administration of CM from HQ-, PDRN-, and PVN-treated UV-B-radiated keratinocytes. The most prominent increasing effect was detected when the supernatant from PVN-treated UV-B-radiated keratinocytes was used for melanocyte treatment (Figure 2A,B). The tyrosinase activity and melanin content significantly increased in melanocytes treated with CM from UV-B-radiated keratinocytes (Figure 2C,D). However, these parameters significantly decreased in response to the administration of CM from HQ-, PDRN-, and PVN-treated UV-B-radiated keratinocytes. The most prominent increasing effect was observed for the CM from PVN-treated keratinocytes.

The expression levels of p53 and MITF in the skin of UV-B-radiated animals were significantly higher than those in the skin of normal control animals (Figure 2E and Supplementary Figure S4). However, the levels significantly decreased in response to the topical application of HQ, PDRN, and PVN. The most prominent decreasing effect was shown for the PVN.

The tyrosinase activity in the skin of UV-B-radiated animals was significantly higher than that of normal control animals (Figure 2F). The tyrosinase activity significantly decreased in response to the topical application of HQ, PDRN, and PVN. The decreasing effect was most prominent for PVN. Melanin accumulation, as evaluated by Fontana–Masson staining, was significantly higher in the skin of UV-B-radiated animals than in that of normal control animals (Figure 2G and upper row in Figure 2H). Melanin accumulation significantly decreased in response to the topical application of HQ, PDRN, and PVN, and the decreasing effect was most prominent for PVN. TEM images also exhibited decreased melanosome after PVN treatment (lower row in Figure 2H).

### 2.4. PVN Decreased the Expression of NF-κB and MMP2/3/9 in Both In Vitro and In Vivo Models of UV-B Radiation

We supposed that keratinocytes were first affected by the topical PVN, and then keratinocytes secreted effective factors that led to changes in fibroblasts. Thus, we applied HQ, PDRN, or PVN to UV-B-radiated keratinocytes and then collected the supernatants of the keratinocyte cultures (Supplementary Figure S2C). Those CMs from keratinocytes were treated with fibroblasts, and the expression of NF-κB and MMP2/3/9 was evaluated in the fibroblasts.

**Figure 3:**
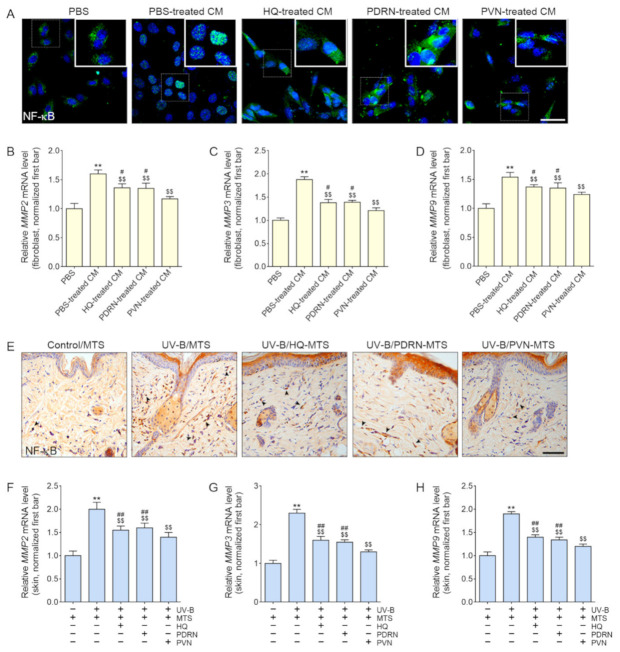
Regulation of NF-κB and MMP2/3/9 expression by HQ, PDRN, and PVN treatment. (**A**) The NF-κB expression in the fibroblast treated with PBS or CM from UV-B-radiated keratinocytes was determined by immunofluorescence staining (scale bar = 50 µm). CM was obtained from UV-B-radiated keratinocyte culture treated with HQ, PDRN, or PVN. (**B**–**D**) The mRNA expression levels of MMP2/3/9 determined in the fibroblast treated with PBS or CM from UV-B-radiated keratinocytes. The mRNA levels were normalized by Actb and expressed relative to the levels in the PBS group. (**E**) The NF-κB expression in the UV-B-radiated animal skin was determined by immunohistochemistry (scale bar = 100 µm). The arrows indicated positive NF-κB expression in the nuclei. (**F**–**H**) The mRNA expression levels of MMP2/3/9 determined in the UV-B-radiated animal skin. The mRNA levels were normalized by Actb and expressed relative to the levels in the control group. Data are presented as the mean ± standard deviation: **, *p* < 0.01 vs. first bar; $$, *p* < 0.01 vs. second bar; #, *p* < 0.05 and ##, *p* < 0.01 vs^.^ 5th bar (Mann–Whitney U test). CM, conditioned medium; HQ, hydroquinone; MMP, matrix metalloproteinase; MTS, microneedling therapy system; NF-κB, nuclear factor kappa-light-chain-enhancer of activated B cells; PBS, phosphate-buffered saline; PDRN, [olydeoxyribonucleotide; PVN, polydeoxyribonucleotide+vitamin C+niacinamide; UV-B, ultraviolet B.

The expression of NF-κB in fibroblasts increased in response to the application of CM from UV-B-radiated keratinocytes (Figure 3A and Supplementary Figure S5A). However, the expression significantly decreased in response to the application of CM from HQ-, PDRN-, and PVN-treated UV-B-radiated keratinocytes.

The mRNA expression of MMP2/3/9 increased in response to the application of CM from UV-B-radiated keratinocytes and significantly decreased in response to the application of CM from HQ-, PDRN-, and PVN-treated UV-B-radiated keratinocytes (Figure 3B–D). The most prominent decreasing effect was shown by CM from PVN-treated UV-B-radiated keratinocytes.

The expression levels of NF-κB and the mRNA expression of MMP2/3/9 were significantly increased in UV-B-radiated animal skin compared with normal control animal skin (Figure 3E–H and Supplementary Figure S5B). These expression levels significantly decreased in response to topical application of HQ, PDRN, and PVN. The most prominent decreasing effect was shown for the PVN.

### 2.5. PVN Increased the Expression of Collagen and Elastin Fibers in the Skin after UV-B Radiation

**Figure 4:**
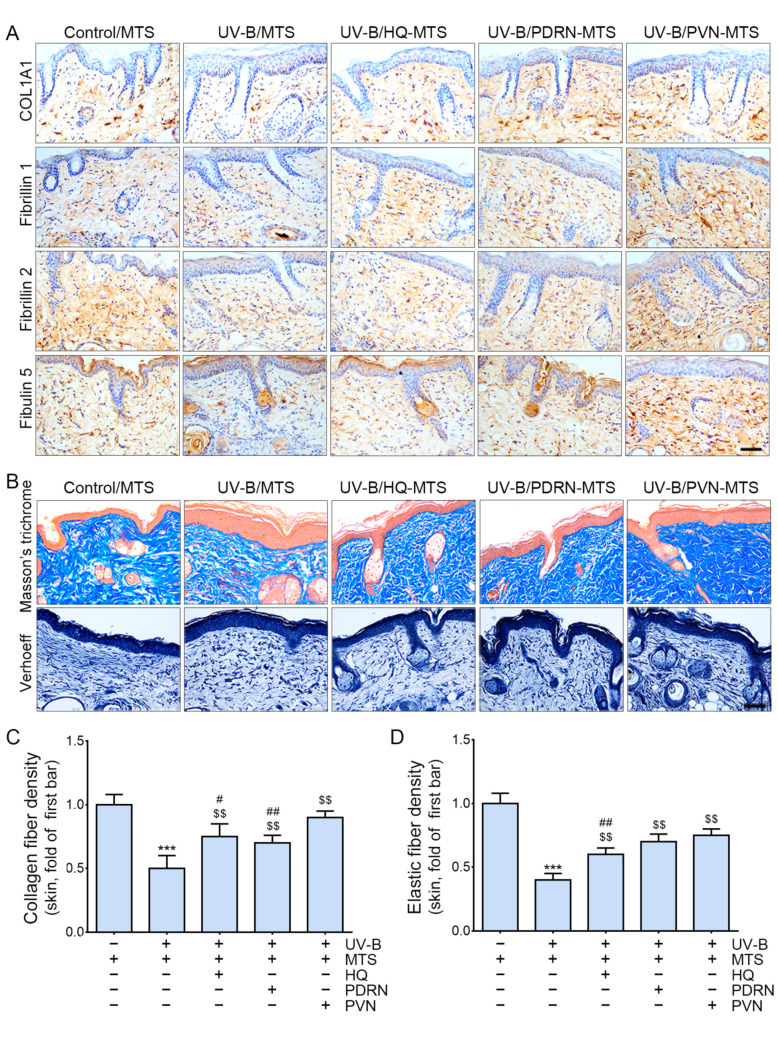
Regulation of the expression of COL1A1, fibrillin 1/2, and fibulin 5 by PVN treatment in UV-B-radiated animal skin. (**A**) COL1A1, fibrillin 1 and 2, and fibulin 5 expression in the UV-B-radiated animal skin was determined by immunohistochemistry (scale bar = 100 µm). (**B**) Collagen and elastin fibers in the UV-B-radiated animal skin were stained for Masson’s trichrome and Verhoeff staining (scale bar = 100 µm). (**C**) The density of collagen fiber was quantified by Masson’s trichrome staining. (**D**) The density of elastin fibers was quantified by Verhoeff staining. Data are presented as the mean ± standard deviation: ***, *p* < 0.001 vs. first bar; $$, *p* < 0.01 vs. second bar; #, *p* < 0.05 and ##, *p* < 0.01 vs. 5th bar (Mann–Whitney U test). COL1A1, collagen Type I α1 chain; HQ, hydroquinone; MTS, microneedling treatment system; PDRN, polydeoxyribonucleotide; PVN, polydeoxyribonucleotide+vitamin C+niacinamide; UV-B, ultraviolet B.

Collagen type I α1 (COL1A1) is the main element of type I collagen [51]; thus, we performed COL1A1 staining to evaluate the expression of type I collagen in the skin. The expression of COL1A1 significantly decreased in response to UV-B and significantly increased in response to topical application of HQ, PDRN, and PVN (Figure 4A and Supplementary Figure S6A). The increasing effect of the PVN was significantly higher than that of HQ.

The expression of fibrillin 1 and 2 significantly decreased in response to UV-B and significantly increased in response to topical application of HQ, PDRN, and PVN (Figure 4A and Supplementary Figure S6B,C). The most prominent increasing effect was observed for PVN. The expression of fibulin 5 significantly decreased in response to UV-B and significantly increased in response to the topical application of HQ, PDRN, and PVN (Figure 4A and Supplementary Figure S6D). The most prominent increasing effect was shown for PVN.

The amount of collagen fiber evaluated by Masson’s trichrome staining significantly decreased in response to UV-B and significantly increased in response to topical application of HQ, PDRN, and PVN (Figure 4B,C). The most prominent increasing effect was shown for PVN.

The amount of elastin fiber evaluated by Verhoeff staining significantly decreased in response to UV-B and significantly increased in response to topical application of HQ, PDRN, and PVN (Figure 4B,D). The most prominent increasing effect was shown for PVN.

## 3. Discussion

UV-induced skin pigmentation causes various cosmetic problems [52]. The main mechanism of melanogenesis by UV is oxidative stress [9,10]. The increased ROS led to the activation of p53, which upregulates α-MSH and MITF [9,10]. Upregulated MITF enhances tyrosinase expression and melanogenesis [9,10]. Thus, many antioxidant agents are expected to decrease melanogenesis by attenuating oxidative stress [43,44]. Vitamin C and niacinamide are representative antioxidant agents that have been evaluated largely for the treatment of hyperpigmentation [43,44]. It is reasonable that a proper combination of those agents as a topical formula would show a more significant effect on decreasing melanogenesis than a single agent. Thus, we evaluated the effects of the combination of PDRN, vitamin C, and niacinamide on attenuating skin hyperpigmentation induced by UV-B radiation. It is known that antioxidant agents lead to upregulation of NRF2, which subsequently increases HO-1 expression [14]. HO-1 is involved in decreasing oxidative stress by decreasing NADPH oxidase and increasing SOD levels [14].

In our study, activated NRF2 and the expression of HO-1 increased in response to HQ, PDRN, and PVN in both UV-B-radiated keratinocyte and animal skin. Moreover, the activity of NADPH oxidase decreased, while the SOD activity increased in response to HQ, PDRN, and PVN in both UV-B-radiated keratinocyte and animal skin. Decreasing NADPH oxidase activity and increasing SOD activity were most prominently detected in response to PVN.

We also evaluated the expression of p53/MITF and the activity of tyrosinase to confirm the effect of the PVN on decreasing UV-B-induced melanogenesis. HQ, PDRN, and PVN were applied topically to animal skin; thus, we assumed that keratinocytes might be more affected by topical agents and that effective factors were secreted by keratinocytes. Thus, keratinocytes affect melanocytes and fibroblasts by paracrine effects.

We designed an in vitro model to evaluate these consumptions. Keratinocytes were radiated with UV-B, and then HQ, PDRN, and PVN were added to the keratinocyte culture medium. The CM from the keratinocyte culture was treated with melanocytes or fibroblasts to evaluate the changes in melanogenesis or collagen/elastin synthesis, respectively. The expression of p53 and MITF decreased in response to HQ, PDRN, and PVN in both the in vitro model and animal skin. The most prominent effect on decreasing tyrosinase activity was shown for the PVN. The melanin content in both melanocytes and animal skin decreased in response to HQ, PDRN, and PVN. The most prominent effect on decreasing melanin deposition was shown for the PVN.

The dermal ECM contains matrix proteins, including elastin, collagen, and proteoglycans, and these ECM proteins play a main role in maintaining the resiliency and strength of the skin. UV-induced skin aging is caused by dermal ECM changes, which are induced by the senescence of fibroblasts and decreased synthesis or increased degradation of dermal collagen fiber [53]. These ECM changes lead to wrinkle formation, fragility of aged skin, and increased laxity [54].

Many reports have shown that UV light activates various MMPs in the skin [55]. MMP-1 is known to degrade type I and type III collagen and leads to complete collagen destruction [56]. MMP-2 degrades type I and type IV collagen [57]. MMP-3 could largely degrade type IV collagen, fibronectin, and proteoglycans. MMP-9 leads to additional degradation of collagen fragments initiated by MMP-1 [56]. Collagen fibers are a main element of the dermis [58]. Approximately 70–80% of the dry weight of the skin is attributed to collagen fiber, which provides mechanical and structural integrity to the dermis [58]. Even though elastic fibers are a less proportional component that comprises 2–4% of the dermal ECM, elastin mainly provides skin elasticity [58]. In addition to collagen synthesis, elastic fiber synthesis is reduced, leading to impaired elasticity during dermal aging [59].

The main component of elastin fiber is elastin, which is composed of tropoelastin [60]. Elastin fiber also contains fibrillin-rich microfibrils, such as fibrillin 1 and fibrillin 2. Both elastin and fibrillin are important to maintain skin elasticity [60]. UV radiation decreases both the levels of elastin and fibrillin and leads to the formation of wrinkles and impaired elasticity [60]. Moreover, fibulin 5, which is an element of microfibrils, is also crucial to the synthesis of elastic fibers [61]. It is also known that UV-radiation-induced increases in MMP-2, -3, -9, -12, and -13 lead to the degradation of fibrillin 1 and fibrillin-rich microfibrils [62]. These MMPs lead to the destruction and remodeling of elastin fibers, thus resulting in wrinkling [63].

NF-κB is one of the main signaling pathways that upregulates MMPs in response to UV radiation [4]. In our study, the expression of NF-κB decreased in response to HQ, PDRN, and PVN in both fibroblasts treated with the CM of UV-B-radiated keratinocytes and animal skin. The expression of MMP-2, MMP-3, and MMP-9 decreased in response to HQ, PDRN, and PVN in fibroblasts treated with UV-B-radiated keratinocyte and animal skin.

The expression of collagen fibers in animal skin was evaluated by COL1A1 staining and Masson’s trichrome staining. The expression and the amount of collagen fibers evaluated with COL1A1 staining and Masson’s trichrome staining significantly decreased in response to UV-B radiation and increased in response to HQ, PDRN, and PVN. The expression of elastin fibers was evaluated via the expression of fibrillin 1, fibrillin 2, and fibulin 5. These expression levels were decreased by UV-B and increased in response to HQ, PDRN, and PVN. The most prominent increasing effect was shown for the PVN. The amount of elastin fiber evaluated by Verhoeff staining was also decreased by UV-B radiation and increased in response to HQ, PDRN, and PVN. It seemed that HQ, PDRN, and PVN decreased collagen and elastin fiber destruction by decreasing MMPs in UV-B-radiated skin. Moreover, HQ, PDRN, and PVN might increase the synthesis of elastin fibers. These effects were most prominent compared with those of individual applications of PDRN or HQ when three compounds, namely PDRN, vitamin C, and niacinamide, were applied to the skin as topical formulas.

Several treatments, such as topical agents, are available for decreasing collagen loss or for collagen-replacement injections to increase the formation of new collagen. However, procedures or agents that can decrease changes in elastin and improve skin elasticity in photoaged skin are rare [64]. Thus, new agents that primarily act on elastin fibers should be identified for use as treatments for impaired skin elasticity. We hypothesize that, by enhancing the synthesis of elastin fibers, topical formulas of PDRN, vitamin C, and niacinamide could have potential as treatments for improving skin elasticity. Moreover, PVN showed decreased skin pigmentation and collagen fiber degradation by MMPs.

Vitamin C shows poor chemical stability in the topical formulations, since it is easily destroyed by oxidation [65]. Moreover, vitamin C exhibits poor skin absorption when it is applied as normal topical formulations that are in the pH range of 5 to 6 for satisfying skin compatibility [66,67]. Topically applied drugs or formulations show poor skin permeability, which is limited by the stratum corneum [68]. Thus, many tried to develop efficient methods to deliver topical drugs or cosmetics, such as vitamin C, across the stratum corneum [69].

The microneedling system or devices are used to increase topical drug delivery [70]. The microneedling system could generate multiple microscopic channels in the skin via needles of 0.5–3 mm in length [71,72]. Therapeutic molecules could be more effectively delivered into the dermis by using temporary skin disruption while generating channels, which could increase the therapeutic response [73,74]. Various microneedling systems have been used to deliver therapeutic molecules, such as vitamin C, various peptides, and retinyl retinoate [74,75,76,77,78,79]. Microneedling systems have been reported to improve scars and wrinkles via a mechanically generated hole in the skin even without adding therapeutic molecules [80]. Generating a hole in the skin is a controlled trauma that enhances the release of various growth factors, enhancing the production of collagen, angiogenesis, and the generation of elastin in the papillary dermis and eventually promoting skin rejuvenation [80,81,82,83,84].

In our study, we used an MTS, supposing this strategy should increase the delivery of HQ, PDRN, and PVN. Even though we could not directly evaluate whether the skin permeabilities of HQ, PDRN, or PVN were increased by using a MTS in the animal skin, all of our study results showed that PVN showed decreasing melanogenesis and increasing collagen and elastin fibers in the UV-B-radiated animal skin. However, direct measurement of the penetration ability of PVN in the skin should be evaluated to improve the delivery of PVN in a future study. We did not evaluate the chemical stability of vitamin C in the PVN, and this is one of the limitations of our study.

**Figure 5:**
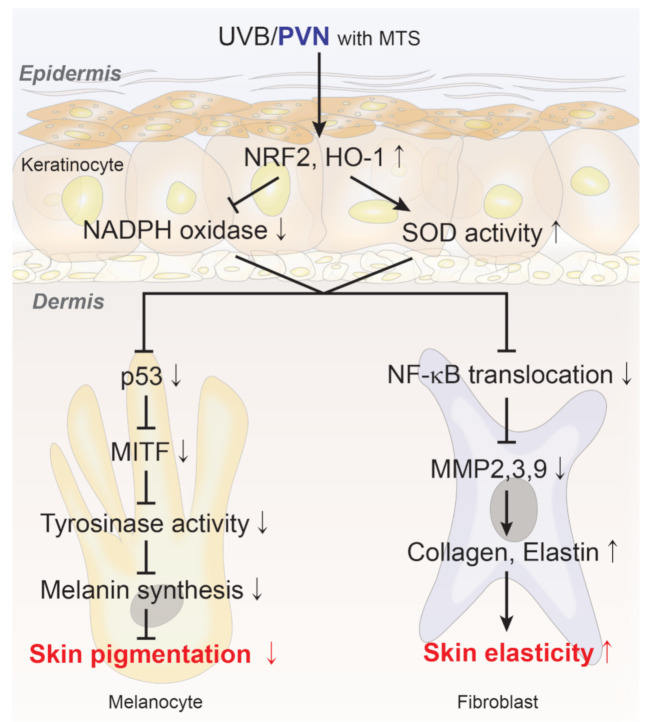
Schematic summary of the effect of PVN on UV-B. HO-1, heme oxygenase-1; HQ, hydroquinone; MITF, microphthalmia-associated transcription factor; MMP, matrix metalloproteinase; MTS, microneedling therapy system; NADPH, nicotinamide-adenine-dinucleotide phosphate hydrogen; NF-κB, nuclear factor kappa-light-chain-enhancer of activated B cells; PDRN, polydeoxyribonucleotide; pNRF2, phosphorylated nuclear factor erythroid-2-related factor 2; PVN, polydeoxyribonucleotide+vitamin C+niacinamide; p53, tumor protein p53; SOD, superoxide dismutase; UV-B, ultraviolet B.

In conclusion, PVN, which is a mixture of PDRN, vitamin C, and niacinamide that was applied topically to skin via a MTS, showed decreased melanin synthesis and accumulation in the skin by decreasing oxidative stress. The PVN increased the expression of collagen and elastin fibers by decreasing MMPs and increasing elastin fiber synthesis in UV-B-radiated skin (Figure 5).

## 4. Materials and Methods

### 4.1. Experimental Model

#### 4.1.1. Cell Culture

Human primary epidermal keratinocyte (HEKn; ATCC, Manassas, VA, USA), human primary epidermal melanocyte (HEMn; ATCC), and human fibroblast (CCD-986sk; Korean Cell Line Bank, Seoul, Korea) cells were used in this study. HEKn and HEMn cells were cultivated with dermal cell basal medium (ATCC) containing a keratinocyte growth kit (ATCC) or melanocyte growth kit (ATCC), respectively. CCD-986sk cells were cultivated with RPMI 1640 medium (Gibco, Grand Island, NY, USA) with 10% fetal bovine serum (Millipore, Burlington, MA, USA) and 1% penicillin/streptomycin (Gibco). All the cells were maintained at 37 °C, under 5% CO_2_.

#### 4.1.2. Preparation of PVN

PVN was prepared just before application as a liquid form. PDRN, niacinamide, and vitamin C were dissolved in distilled water by using a high-speed mixer (T.K. Homo Disper, Model 2.5, PRIMIX, Hyogo, Japan) at 3000 rpm. After complete mixing, PVN was filtered with 0.2 μm filter (S2GPU11RE, Merck, CA, USA) to remove bacteria. There was no sediment in the solution of PVN when it was applied. A total of 1 mM of PVN contains 0.55 mM of niacinamide, 0.25 mM of vitamin C, and 0.18 mM of PDRN in liquid form.

#### 4.1.3. In Vitro Models

First, to examine the effect of PVN on keratinocytes, HEKn cells were radiated to UV-B (200 mJ/cm^2^) for 5 min before HQ (100 µM), PDRN (1 mM), and PVN (1 mM) application and incubated for 24 h at 37 °C under 5% CO_2_ (Supplementary Figure S2A).

In order to confirm whether melanocytes were affected by PVN-treated keratinocytes, HQ, PDRN, or PVN was added to UV-B-radiated HEKn cells, and then the supernatant from those cell culture was collected after 24 h. Those CMs from HEKn cells’ culture or PBS were subsequently treated with HEMn cells and incubated for 24 h at 37 °C under 5% CO_2_ (Supplementary Figure S2B).

Additionally, HQ, PDRN, or PVN was added to UV-B-radiated HEKn cells, and then the supernatant from those cell culture was harvested after 24 h. Subsequently, those CMs from HEKn cells cultures or PBS were treated with CCD-986sk cells and cultivated for 24 h at 37 °C under 5% CO_2_ to determine whether there was a change in fibroblasts by PVN-treated keratinocytes (Supplementary Figure S2C).

#### 4.1.4. UV-B-Radiated Mice Model

HRM-2 mice (female, 5 weeks old, 20–25 g) were obtained from SLC Inc. (Shizuoka, Japan) and reared for 2 weeks. The mice were housed in cages under a controlled temperature of 23 °C with a 12 h light/dark cycle and free access to food and water. After the adaptation period, the mice were randomly divided into five groups as follows: (1) control (distilled water with MTS), (2) UV-B (UV-B radiation and then distilled water with MTS), (3) UV-B/HQ (UV-B radiation and then hydroquinone treatment with MTS), (4) UV-B/PDRN (UV-B radiation and then polydeoxyribonucleotide treatment with MTS), and (5) UV-B/PVN (UV-B radiation and then mixture comprising PDRN, vitamin C, and niacinamide treatment with MTS). The mice were radiated with UV-B at 200 mJ/cm^2^. UV-B was measured every 2 days for 5 min for 10 days and then for 5 min every day for the next 3 days (total 13 days) [85]. Subsequently, the mice were treated with 50 µL of compound per square centimeter of area with MTS (LARCO-STAMP-02, 16.5 cm in height, 4.5 cm in width, 4.5 cm in vertical, 0.75 mm in needle length, 140 needles, L’arcobaleno, Seoul, Korea). The control and UV-B groups were treated with MTS at 7-day intervals. They were then additionally radiated with UV-B at 2-day intervals for 28 days. This study was approved by the Center of Animal Care and Use Ethics Board of Gachon University (Approval Number LCDI-2021-0068) and executed in accordance with the Institutional Animal Care and Use Committee.

### 4.2. Sample Preparation

#### 4.2.1. RNA Extraction and cDNA Synthesis

The total RNA of cells and frozen skin tissues was extracted by using RNAiso Plus (Takara, Kyoto, Japan) according to the manufacturer’s instructions. The quality and concentration of extracted RNA were confirmed by a Nanodrop 2000 spectrophotometer (Thermo Fisher Scientific, Waltham, MA, USA), and cDNA was synthesized by using a PrimeScript First Strand cDNA Synthesis Kit (Takara) according to the manufacturer’s instructions.

#### 4.2.2. Protein Isolation

The proteins were isolated from the cells and skin tissue by using the EzRIPA lysis kit (ATTO Corporation, Tokyo, Japan). First, the cells and skin tissue were lysed with EzRIPA buffer containing protease and phosphatase inhibitors. Then, the homogenized samples were sonicated and centrifuged at 14,000× *g* for 20 min at 4 °C. Then, the supernatants were transferred to a new tube, and the protein was quantified by using a bicinchoninic acid assay kit (BCA kit; Thermo Fisher Scientific, Waltham, MA, USA).

#### 4.2.3. Paraffin-Embedded Tissue

The harvested skin tissues were fixed with cold 4% paraformaldehyde (PFA; Sigma-Aldrich, cat. 16005, St. Louis, MO, USA) in PBS at 4 °C for 24 h. The fixed skin tissues were washed for 30 min, and a paraffin block was made by using a tissue processor (Thermo Fisher Scientific, Waltham, MA, USA). The paraffin blocks were sectioned to 7 µm in thickness by using a microtome (Leica, Wetzlar, Germany) and dried at 37 °C, overnight, to attach to the slides. The paraffin blocks were passed through xylene and four concentrations of ethanol (i.e., 100, 95, 80, and 70%) to prepare them for staining.

### 4.3. Melanin Content Assays

To assess the melanin content in HEMn cells, CM from HEKn cell culture or PBS was applied to the cells for 24 h. Then, the cells were harvested by trypsin-EDTA and centrifuged at 12,000× *g* for 20 min. After centrifugation, the supernatants were discarded and the pellets were dissolved in 100 µL of 10% dimethyl sulfoxide and 1 N NaOH solution for 20 min at 95 °C. After 20 min, the samples were loaded into 96-well plates, and their absorbance at 490 nm was read by a microplate reader (Molecular Devices).

### 4.4. Immunocytochemistry

To measure the expression level of NF-κB in CM from HEKn cell cultures or PBS-treated CCD-986sk cells, the cells were seeded at 3 × 10^5^ cells/well in 8-well chamber slides (Nunc, Waltham, MA, USA) for 12 h. After applying CM from HEKn cell cultures or PBS for 24 h, the cells were washed with PBS. Then the cells were fixed with cold-4% PFA for 5 min and washed with PBS. The slides were treated with normal serum for 1 h at room temperature to reduce nonspecific antigen–antibody interactions. NF-κB primary antibodies were incubated (listed in Supplementary Table S1) for 24 h at 4 °C and then washed with PBS. Then the slides were loaded with secondary antibody (Alexa Fluor 488; Invitrogen, Waltham, MA, USA) for 1 h at room temperature and rinsed with PBS. After washing with PBS, the samples were stained with 4′,6-diamidino-2-phenylindole (DAPI; Sigma-Aldrich, Burlington, MA, USA) solution for 30 s at room temperature to stain the nuclei, rinsed with PBS, and then mounted by using a vector shield solution (Vector Laboratories, Burlingame, CA, USA). The slides were imaged with a confocal microscope (LSM 710, Carl Zeiss, Oberkochen, Germany), and the images were analyzed with Zen 2009 software (Carl Zeiss).

### 4.5. Enzyme-Linked Immunosorbent Assay (ELISA)

NADPH oxidase (Abcam, Waltham, MA, USA) and superoxide dismutase (SOD; Abcam) activity in the UV-B-radiated HEKn cells and skin tissue of each group were determined by using appropriate kits, following the manufacturers’ instructions.

Tyrosinase activity (Abcam) in the UV-B- or compound-conditioned medium-treated HEMn cells and skin tissue of each group was determined by using appropriate kits, following the manufacturers’ instructions.

### 4.6. Western Blotting

A total of 30 μg of isolated skin proteins was separated on 8–12% polyacrylamide gels and transferred to polyvinylidene fluoride membranes (Millipore, Burlington, MA, USA) by a power station (ATTO, Osaka, Japan). After blocking using 5% skim milk and washing with Tris-buffered saline with 0.1% Tween 20 (TTBS), we incubated the membranes with primary antibodies (Supplementary Table S1) for 12 h at 4 °C and then washed with TTBS. The membranes were then incubated with a secondary antibody (Vector Laboratories, Burlingame, CA, USA) and rinsed with TTBS. Subsequently, an enhanced chemiluminescence detection reagent (GE Healthcare, Chicago, IL, USA) was used to visualize the immunoreactive proteins on the membrane.

### 4.7. Immunohistochemistry

Skin paraffin blocks made by using a tissue processor (Thermo Fisher Scientific, Waltham, MA, USA) were sectioned to 7 µm, using a microtome (Leica, Wetzlar, Germany), and incubated at 37 °C, overnight, to maintain their attachment to the slides. The sectioned slides were passed through xylene and four concentrations of ethanol (i.e., 100%, 95%, 80%, and 70%) to remove the paraffin for staining.

The sectioned skin-tissue slides were incubated in 3% hydrogen peroxide in methanol for 30 min at room temperature to block endogenous peroxidase. The tissue slides were subsequently washed by using PBS and then incubated together with primary antibodies (Supplementary Table S1) in normal serum for 12 h at 4 °C. The slides were rinsed with PBS and then incubated with a biotinylated secondary antibody, using an ABC kit (Vector Laboratories, Inc., Burlingame, CA, USA), for 2 h at room temperature. After washing with PBS, the tissue slides were developed by using 3,3′-diaminobenzidine (Sigma-Aldrich) for 15 min, until confirmation of brown signals. To identify nuclei, the tissue slides were stained in hematoxylin solution for 1 min and then mounted with DPX mounting solution (Sigma-Aldrich). Images of the stained tissues were taken under an optical microscope (Olympus Optical Co., Tokyo, Japan) and analyzed by using ImageJ software (NIH, Bethesda, MD, USA).

### 4.8. Quantitative Real-Time Polymerase Chain Reaction (qRT-PCR)

The qRT-PCR reagent mixture, consisting of SYBR Green reagent (Takara), 1 µg of synthesized cDNA template, and a 10 pmol of primer (Supplementary Table S2), was added to a 384-well multi-plate and then analyzed by a CFX386 Touch Real-Time PCR System (Bio-Rad, Hercules, CA, USA).

### 4.9. Histological Analysis

#### 4.9.1. Fontana–Masson Staining

The skin tissues were incubated in Fontana ammoniacal silver solution (ScyTek, FMS-1, West Logan, UT, USA) for 30 min at 60 °C, rinsed three times with distilled water, and then incubated in 0.2% gold chloride solution for 30 s. Afterward, the tissues were washed in distilled water and 5% sodium thiosulfate solution for 1 min, counterstained with nuclear fast red stain for 5 min, washed in distilled water, dehydrated in absolute alcohol, and mounted for observations.

#### 4.9.2. Masson Trichrome Staining

The skin tissues were incubated in Bouin’s fluid solution (ScyTek, TRM-2; West Logan, UT, USA) for 1 h, at 60 °C; rinsed three times with distilled water; and then incubated in a working weight solution of iron hematoxylin for 5 min, Biebrich scarlet acid fuchsin solution for 5 min, phosphomolybdic–phosphotungstic acid solution for 12 min, and aniline blue solution for 3 min. Afterward, the tissues were washed in distilled water, dehydrated in absolute alcohol, and mounted for observation. MT-stained fibrosis areas were measured by using ImageJ software [86].

#### 4.9.3. Verhoeff Staining

The skin tissues were incubated in working elastic stain solution (ScyTek, ETS-1, West Logan, UT, USA) for 15 min at room temperature, rinsed three times with tap water, and then incubated in 20 drops of 2% ferric chloride differentiation solution. Afterward, the tissues were washed in distilled water, dehydrated in absolute alcohol, and mounted for observation. The elastin fiber areas were measured by using ImageJ software [87].

### 4.10. Transmission Electron Microscopy Analysis (TEM)

Specimens were fixed for 12 h in 2% glutaraldehyde/2% paraformaldehyde in 0.1 M phosphate buffer (pH 7.4), washed in 0.1 M phosphate buffer, postfixed with 1% OsO4 in 0.1 M phosphate buffer for 2 h, dehydrated in an ascending ethanol series (50%, 60%, 70%, 80%, 90%, 95%, 100%, and 100%) for 10 min each, and infiltrated with propylene oxide for 10 min. The fixed samples were embedded by using a Poly/Bed 812 kit (Polysciences, Warrington, PA, USA) and polymerized in an electron microscope chamber (Dosaka, Katsumi, Japan) at 65 °C for 12 h. The block was equipped with a diamond-equipped ultramicrotome, cut into 200 nm sections, and then stained with toluidine blue for optical microscopy.

The region of interest was then cut into 80 nm sections, using an ultramicrotome; placed on copper grids; double stained with 3% uranyl acetate for 30 min, followed by 3% lead citrate for 7 min; and observed under a transmission electron microscope (JEOL, Tokyo, Japan) equipped with a Megaview III charge-coupled device (CCD) camera (Soft Imaging System, Germany), at an acceleration voltage of 80 kV.

### 4.11. Statistical Analysis

To validate the significance of the difference among the mice, we performed a D’Agostino–Pearson omnibus normality test and then a Kruskal–Wallis test for comparisons of the five groups, followed by a Mann–Whitney U test as a post hoc test. The dam study was validated by an unpaired t-test. All the results are presented as the means ± standard deviations. The entire statistical analysis was performed by using SPSS version 22 (IBM Corporation; Armonk, NY, USA), and the means denoted by different letters indicate significant intergroup differences.

## Data Availability

All data are contained within the article.

## Supplementary Materials

The following supporting information can be downloaded online. Table S1: List of antibodies for Western blotting (WB), immunohistochemistry (IHC), and immunocytochemistry (ICC). Table S2: List of primer for quantitative real time polymerase chain reaction (qRT-PCR). Figure S1: Regulation of melanin amount ratio by various combinations of niacinamide, vitamin C, and PDRN in the α-MSH-treated human melanocytes. Figure S2: Schematic diagram of the in vitro experiment used in this study. Figure S3: Regulation effect on the expression of NRF2 and HO-1 by HQ, PDRN, and PVN treatment in the UV-B-radiated human keratinocytes and animal skin. Figure S4: Regulation of the expression of p53 and MITF by HQ, PDRN, and PVN treatment in UV-B-radiated animal skin. Figure S5: Regulation of the expression of NF-κB by HQ, PDRN, and PVN treatment in the CM-treated human fibroblasts and animal skin. Figure S6: Regulation of the expression of COL1A1, fibrillin 1/2, and fibulin 5 by PVN treatment in UV-B-radiated animal skin.

## Sample Availability

All samples are available from the authors.

## References

[1] Petersen M.J., Hansen C., Craig S. (1992). Ultraviolet A irradiation stimulates collagenase production in cultured human fibroblasts. J. Invest. Derm..

[2] Imokawa G., Ishida K. (2015). Biological mechanisms underlying the ultraviolet radiation-induced formation of skin wrinkling and sagging I: Reduced skin elasticity, highly associated with enhanced dermal elastase activity, triggers wrinkling and sagging. Int. J. Mol. Sci.

[3] Pittayapruek P., Meephansan J., Prapapan O., Komine M., Ohtsuki M. (2016). Role of Matrix Metalloproteinases in Photoaging and Photocarcinogenesis. Int. J. Mol. Sci.

[4] Cannarozzo G., Fazia G., Bennardo L., Tamburi F., Amoruso G.F., Del Duca E., Nisticò S.P. (2021). A New 675 nm Laser Device in the Treatment of Facial Aging: A Prospective Observational Study. Photobiomodul. Photomed. Laser Surg..

[5] Nistico S.P., Silvestri M., Zingoni T., Tamburi F., Bennardo L., Cannarozzo G. (2021). Combination of Fractional CO_2_ Laser and Rhodamine-Intense Pulsed Light in Facial Rejuvenation: A Randomized Controlled Trial. Photobiomodul. Photomed. Laser Surg..

[6] Wlaschek M., Maity P., Makrantonaki E., Scharffetter-Kochanek K. (2021). Connective Tissue and Fibroblast Senescence in Skin Aging. J. Invest. Derm..

[7] Park J.E., Pyun H.B., Woo S.W., Jeong J.H., Hwang J.K. (2014). The protective effect of Kaempferia parviflora extract on UVB-induced skin photoaging in hairless mice. Photodermatol. Photoimmunol. Photomed..

[8] Rani S., Kumar R., Kumarasinghe P., Bhardwaj S., Srivastava N., Madaan A., Parsad D. (2018). Melanocyte abnormalities and senescence in the pathogenesis of idiopathic guttate hypomelanosis. Int. J. Derm..

[9] Costin G.E., Hearing V.J. (2007). Human skin pigmentation: Melanocytes modulate skin color in response to stress. FASEB J..

[10] Khlgatian M.K., Hadshiew I.M., Asawanonda P., Yaar M., Eller M.S., Fujita M., Norris D.A., Gilchrest B.A. (2002). Tyrosinase gene expression is regulated by p53. J. Invest. Derm..

[11] Nahhas A.F., Mohammad T.F., Hamzavi I.H. (2017). Vitiligo Surgery: Shuffling Melanocytes. J. Investig. Derm. Symp. Proc..

[12] Huggins R.H., Henderson M.D., Mulekar S.V., Ozog D.M., Kerr H.A., Jabobsen G., Lim H.W., Hamzavi I.H. (2012). Melanocyte-keratinocyte transplantation procedure in the treatment of vitiligo: The experience of an academic medical center in the United States. J. Am. Acad. Derm..

[13] Deng Y., Zhu J., Mi C., Xu B., Jiao C., Li Y., Xu D., Liu W., Xu Z. (2015). Melatonin antagonizes Mn-induced oxidative injury through the activation of keap1-Nrf2-ARE signaling pathway in the striatum of mice. Neurotox Res..

[14] Kim J.K., Jang H.D. (2014). Nrf2-mediated HO-1 induction coupled with the ERK signaling pathway contributes to indirect antioxidant capacity of caffeic acid phenethyl ester in HepG2 cells. Int. J. Mol. Sci.

[15] Jiang F., Roberts S.J., Datla S.r., Dusting G.J. (2006). NO modulates NADPH oxidase function via heme oxygenase-1 in human endothelial cells. Hypertension.

[16] Marrot L., Jones C., Perez P., Meunier J.R. (2008). The significance of Nrf2 pathway in (photo)-oxidative stress response in melanocytes and keratinocytes of the human epidermis. Pigment. Cell Melanoma Res..

[17] Kokot A., Metze D., Mouchet N., Galibert M.D., Schiller M., Luger T.A., Böhm M. (2009). Alpha-melanocyte-stimulating hormone counteracts the suppressive effect of UVB on Nrf2 and Nrf-dependent gene expression in human skin. Endocrinology.

[18] Draelos Z.D. (2007). Skin lightening preparations and the hydroquinone controversy. Derm. Ther..

[19] Yanagishita-Nakatsuji S., Fukai K., Ohyama A., Umekoji A., Sowa-Osako J., Tsuruta D. (2017). Probable allergic contact dermatitis from hydroquinone presenting as leukomelanoderma: Report of two cases. J. Derm..

[20] Mishra S.N., Dhurat R.S., Deshpande D.J., Nayak C.S. (2013). Diagnostic utility of dermatoscopy in hydroquinone-induced exogenous ochronosis. Int. J. Derm..

[21] Findlay G.H. (1982). Ochronosis following skin bleaching with hydroquinone. J. Am. Acad. Derm..

[22] Kari F.W., Bucher J., Eustis S.L., Haseman J.K., Huff J.E. (1992). Toxicity and carcinogenicity of hydroquinone in F344/N rats and B6C3F1 mice. Food Chem. Toxicol..

[23] Tsutsui T., Hayashi N., Maizumi H., Huff J., Barrett J.C. (1997). Benzene-, catechol-, hydroquinone- and phenol-induced cell transformation, gene mutations, chromosome aberrations, aneuploidy, sister chromatid exchanges and unscheduled DNA synthesis in Syrian hamster embryo cells. Mutat Res..

[24] Yu M.H., Lee S.O. (2016). Hydroquinone stimulates cell invasion through activator protein-1-dependent induction of MMP-9 in HepG2 human hepatoma cells. Food Chem. Toxicol..

[25] Saade D.S., Maymone M.B.C., De La Garza H., Secemsky E.A., Kennedy K.F., Vashi N.A. (2021). Trends in Use of Prescription Skin Lightening Creams. Int. J. Environ. Res. Public Health.

[26] Sheth V.M., Pandya A.G. (2011). Melasma: A comprehensive update: Part II. J. Am. Acad. Derm..

[27] Dlova N.C., Hamed S.H., Tsoka-Gwegweni J., Grobler A. (2015). Skin lightening practices: An epidemiological study of South African women of African and Indian ancestries. Br. J. Derm..

[28] Galeano M., Bitto A., Altavilla D., Minutoli L., Polito F., Calò M., Lo Cascio P., Stagno d’Alcontres F., Squadrito F. (2008). Polydeoxyribonucleotide stimulates angiogenesis and wound healing in the genetically diabetic mouse. Wound Repair Regen..

[29] Squadrito F., Bitto A., Irrera N., Pizzino G., Pallio G., Minutoli L., Altavilla D. (2017). Pharmacological Activity and Clinical Use of PDRN. Front. Pharm..

[30] An J., Park S.H., Ko I.G., Jin J.J., Hwang L., Ji E.S., Kim S.H., Kim C.J., Park S.Y., Hwang J.J. (2017). Polydeoxyribonucleotide Ameliorates Lipopolysaccharide-Induced Lung Injury by Inhibiting Apoptotic Cell Death in Rats. Int. J. Mol. Sci..

[31] Lee D.W., Hyun H., Lee S., Kim S.Y., Kim G.T., Um S., Hong S.O., Chun H.J., Yang D.H. (2019). The Effect of Polydeoxyribonucleotide Extracted from Salmon Sperm on the Restoration of Bisphosphonate-Related Osteonecrosis of the Jaw. Mar. Drugs.

[32] Shin D.Y., Park J.U., Choi M.H., Kim S., Kim H.E., Jeong S.H. (2020). Polydeoxyribonucleotide-delivering therapeutic hydrogel for diabetic wound healing. Sci. Rep..

[33] Jeong W., Yang C.E., Roh T.S., Kim J.H., Lee J.H., Lee W.J. (2017). Scar Prevention and Enhanced Wound Healing Induced by Polydeoxyribonucleotide in a Rat Incisional Wound-Healing Model. Int. J. Mol. Sci..

[34] Kwon D.R., Park G.Y., Lee S.C. (2018). Treatment of Full-Thickness Rotator Cuff Tendon Tear Using Umbilical Cord Blood-Derived Mesenchymal Stem Cells and Polydeoxyribonucleotides in a Rabbit Model. Stem Cells Int..

[35] Thellung S., Florio T., Maragliano A., Cattarini G., Schettini G. (1999). Polydeoxyribonucleotides enhance the proliferation of human skin fibroblasts: Involvement of A2 purinergic receptor subtypes. Life Sci..

[36] Bigliardi P. (1982). Treatment of acute radiodermatitis of first and second degrees with semi-greasy placenta ointment. Int. J. Tissue React..

[37] Belletti S., Uggeri J., Gatti R., Govoni P., Guizzardi S. (2007). Polydeoxyribonucleotide promotes cyclobutane pyrimidine dimer repair in UVB-exposed dermal fibroblasts. Photodermatol. Photoimmunol. Photomed..

[38] Philips N., Auler S., Hugo R., Gonzalez S. (2011). Beneficial regulation of matrix metalloproteinases for skin health. Enzym. Res..

[39] Labat-Robert J., Fourtanier A., Boyer-Lafargue B., Robert L. (2000). Age dependent increase of elastase type protease activity in mouse skin. Effect of UV-irradiation. J. Photochem. Photobiol. B.

[40] Kim Y.J., Kim M.J., Kweon D.K., Lim S.T., Lee S.J. (2020). Polydeoxyribonucleotide Activates Mitochondrial Biogenesis but Reduces MMP-1 Activity and Melanin Biosynthesis in Cultured Skin Cells. Appl. Biochem. Biotechnol..

[41] Porsch H., Bernert B., Mehić M., Theocharis A.D., Heldin C.H., Heldin P. (2013). Efficient TGFβ-induced epithelial-mesenchymal transition depends on hyaluronan synthase HAS2. Oncogene.

[42] Kruglikov I.L., Scherer P.E. (2016). Dermal adipocytes and hair cycling: Is spatial heterogeneity a characteristic feature of the dermal adipose tissue depot?. Exp. Derm..

[43] Telang P.S. (2013). Vitamin C in dermatology. Indian Derm. Online J..

[44] Chhabra G., Garvey D.R., Singh C.K., Mintie C.A., Ahmad N. (2019). Effects and Mechanism of Nicotinamide Against UVA- and/or UVB-mediated DNA Damages in Normal Melanocytes. Photochem. Photobiol..

[45] Ratcliffe D.R., Iqbal J., Hussain M.M., Cramer E.B. (2009). Fibrillar collagen type I stimulation of apolipoprotein B secretion in Caco-2 cells is mediated by beta1 integrin. Biochim. Biophys. Acta.

[46] Philips N., Chalensouk-Khaosaat J., Gonzalez S. (2018). Simulation of the Elastin and Fibrillin in Non-Irradiated or UVA Radiated Fibroblasts, and Direct Inhibition of Elastase or Matrix Metalloptoteinases Activity by Nicotinamide or Its Derivatives. J. Cosmet. Sci..

[47] Virador V.M., Kobayashi N., Matsunaga J., Hearing V.J. (1999). A standardized protocol for assessing regulators of pigmentation. Anal. Biochem..

[48] Lei T.C., Virador V.M., Vieira W.D., Hearing V.J. (2002). A melanocyte-keratinocyte coculture model to assess regulators of pigmentation in vitro. Anal. Biochem..

[49] Hakozaki T., Minwalla L., Zhuang J., Chhoa M., Matsubara A., Miyamoto K., Greatens A., Hillebrand G.G., Bissett D.L., Boissy R.E. (2002). The effect of niacinamide on reducing cutaneous pigmentation and suppression of melanosome transfer. Br. J. Derm..

[50] Greatens A., Hakozaki T., Koshoffer A., Epstein H., Schwemberger S., Babcock G., Bissett D., Takiwaki H., Arase S., Wickett R.R. (2005). Effective inhibition of melanosome transfer to keratinocytes by lectins and niacinamide is reversible. Exp. Derm..

[51] Ma H.P., Chang H.L., Bamodu O.A., Yadav V.K., Huang T.Y., Wu A., Yeh C.T., Tsai S.H., Lee W.H. (2019). Collagen 1A1 (COL1A1) Is a Reliable Biomarker and Putative Therapeutic Target for Hepatocellular Carcinogenesis and Metastasis. Cancers.

[52] Kalasho B.D., Minokadeh A., Zhang-Nunes S., Zoumalan R.A., Shemirani N.L., Waldman A.R., Pletzer V., Zoumalan C.I. (2020). Evaluating the Safety and Efficacy of a Topical Formulation Containing Epidermal Growth Factor, Tranexamic Acid, Vitamin C, Arbutin, Niacinamide and Other Ingredients as Hydroquinone 4% Alternatives to Improve Hyperpigmentation: A Prospective, Randomized, Controlled Split Face Study. J. Cosmet. Sci..

[53] Murakami H., Shimbo K., Inoue Y., Takino Y., Kobayashi H. (2012). Importance of amino acid composition to improve skin collagen protein synthesis rates in UV-irradiated mice. Amino Acids.

[54] Fisher G.J., Kang S., Varani J., Bata-Csorgo Z., Wan Y., Datta S., Voorhees J.J. (2002). Mechanisms of photoaging and chronological skin aging. Arch. Derm..

[55] Lu J., Guo J.H., Tu X.L., Zhang C., Zhao M., Zhang Q.W., Gao F.H. (2016). Tiron Inhibits UVB-Induced AP-1 Binding Sites Transcriptional Activation on MMP-1 and MMP-3 Promoters by MAPK Signaling Pathway in Human Dermal Fibroblasts. PLoS ONE.

[56] Quan T., Qin Z., Xia W., Shao Y., Voorhees J.J., Fisher G.J. (2009). Matrix-degrading metalloproteinases in photoaging. J. Investig. Derm. Symp. Proc..

[57] Illman S.A., Keski-Oja J., Lohi J. (2001). Promoter characterization of the human and mouse epilysin (MMP-28) genes. Gene.

[58] Uitto J. (1979). Biochemistry of the elastic fibers in normal connective tissues and its alterations in diseases. J. Invest. Derm..

[59] Uehara E., Hokazono H., Hida M., Sasaki T., Yoshioka H., Matsuo N. (2017). GABA promotes elastin synthesis and elastin fiber formation in normal human dermal fibroblasts (HDFs). Biosci. Biotechnol. Biochem..

[60] Kielty C.M., Sherratt M.J., Shuttleworth C.A. (2002). Elastic fibres. J. Cell Sci..

[61] Nakamura T., Lozano P.R., Ikeda Y., Iwanaga Y., Hinek A., Minamisawa S., Cheng C.F., Kobuke K., Dalton N., Takada Y. (2002). Fibulin-5/DANCE is essential for elastogenesis in vivo. Nature.

[62] Ashworth J.L., Murphy G., Rock M.J., Sherratt M.J., Shapiro S.D., Shuttleworth C.A., Kielty C.M. (1999). Fibrillin degradation by matrix metalloproteinases: Implications for connective tissue remodelling. Biochem. J..

[63] Fisher G.J., Datta S.C., Talwar H.S., Wang Z.Q., Varani J., Kang S., Voorhees J.J. (1996). Molecular basis of sun-induced premature skin ageing and retinoid antagonism. Nature.

[64] Ménasche M., Jacob M.P., Godeau G., Robert A.M., Robert L. (1981). Pharmacological studies on elastin peptides (kappa-elastin). Blood clearance, percutaneous penetration and tissue distribution. Pathol. Biol..

[65] Iliopoulos F., Sil B.C., Moore D.J., Lucas R.A., Lane M.E. (2019). 3-O-ethyl-l-ascorbic acid: Characterisation and investigation of single solvent systems for delivery to the skin. Int. J. Pharm X.

[66] Stamford N.P. (2012). Stability, transdermal penetration, and cutaneous effects of ascorbic acid and its derivatives. J. Cosmet. Derm..

[67] Pinnell S.R., Yang H., Omar M., Monteiro-Riviere N., De Buys H.V., Walker L.C., Wang Y., Levine M. (2001). Topical L-ascorbic acid: Percutaneous absorption studies. Derm. Surg..

[68] Pullar J.M., Carr A.C., Vissers M.C.M. (2017). The Roles of Vitamin C in Skin Health. Nutrients.

[69] Sawutdeechaikul P., Kanokrungsee S., Sahaspot T., Thadvibun K., Banlunara W., Limcharoen B., Sansureerungsikul T., Rutwaree T., Oungeun M., Wanichwecharungruang S. (2021). Detachable dissolvable microneedles: Intra-epidermal and intradermal diffusion, effect on skin surface, and application in hyperpigmentation treatment. Sci. Rep..

[70] Chun H.S., Song H.S. (2021). Analysis of Trend of Studies on Microneedle Treatment System (MTS). J. Pharmacopunct..

[71] Prausnitz M.R. (2004). Microneedles for transdermal drug delivery. Adv. Drug Deliv. Rev..

[72] Al-Qallaf B., Das D.B. (2009). Optimizing microneedle arrays for transdermal drug delivery: Extension to non-square distribution of microneedles. J. Drug Target..

[73] Sharma D. (2018). Microneedles: An approach in transdermal drug delivery: A Review. Pharma Tutor..

[74] Waghule T., Singhvi G., Dubey S.K., Pandey M.M., Gupta G., Singh M., Dua K. (2019). Microneedles: A smart approach and increasing potential for transdermal drug delivery system. Biomed. Pharm..

[75] Mohammed Y.H., Yamada M., Lin L.L., Grice J.E., Roberts M.S., Raphael A.P., Benson H.A., Prow T.W. (2014). Microneedle enhanced delivery of cosmeceutically relevant peptides in human skin. PLoS ONE.

[76] Bora P., Kumar L., Bansal A.K. (2008). Microneedle technology for advanced drug delivery: Evolving vistas. Dep. Pharm. Technol. NIPER CRIPS.

[77] Larraneta E., Lutton R.E., Woolfson A.D., Donnelly R.F. (2016). Microneedle arrays as transdermal and intradermal drug delivery systems: Materials science, manufacture and commercial development. Mater. Sci. Eng. R Rep..

[78] Serrano G., Almudéver P., Serrano J.M., Cortijo J., Faus C., Reyes M., Expósito I., Torrens A., Millán F. (2015). Microneedling dilates the follicular infundibulum and increases transfollicular absorption of liposomal sepia melanin. Clin. Cosmet. Investig. Derm..

[79] Zhao Z., Chen Y., Shi Y. (2020). Microneedles: A potential strategy in transdermal delivery and application in the management of psoriasis. RSC Adv..

[80] Aust M.C., Fernandes D., Kolokythas P., Kaplan H.M., Vogt P.M. (2008). Percutaneous collagen induction therapy: An alternative treatment for scars, wrinkles, and skin laxity. Plast. Reconstr. Surg..

[81] Fernandes D., Signorini M. (2008). Combating photoaging with percutaneous collagen induction. Clin. Derm..

[82] El-Domyati M., Barakat M., Awad S., Medhat W., El-Fakahany H., Farag H. (2015). Multiple microneedling sessions for minimally invasive facial rejuvenation: An objective assessment. Int. J. Derm..

[83] Hogan S., Velez M.W., Ibrahim O. (2017). Microneedling: A new approach for treating textural abnormalities and scars. Semin. Cutan. Med. Surg..

[84] McCrudden M.T., McAlister E., Courtenay A.J., González-Vázquez P., Singh T.R., Donnelly R.F. (2015). Microneedle applications in improving skin appearance. Exp. Derm..

[85] Chung K.W., Jeong H.O., Jang E.J., Choi Y.J., Kim D.H., Kim S.R., Lee K.J., Lee H.J., Chun P., Byun Y. (2013). Characterization of a small molecule inhibitor of melanogenesis that inhibits tyrosinase activity and scavenges nitric oxide (NO). Biochim. Biophys. Acta.

[86] Chen Y., Yu Q., Xu C.B. (2017). A convenient method for quantifying collagen fibers in atherosclerotic lesions by ImageJ software. Int. J. Clin. Exp. Med..

[87] Faucz L.L., Will S.E., Rodrigues C.J., Hesse H., Moraes A.C., Maria D.A. (2018). Quantitative evaluation of collagen and elastic fibers after intense pulsed light treatment of mouse skin. Lasers. Surg. Med..

