# Supplementary material for: A Mixture of Topical Forms of Polydeoxyribonucleotide, Vitamin C, and Niacinamide Attenuated Skin Pigmentation and Increased Skin Elasticity by Modulating Nuclear Factor Erythroid 2-like 2
Source: Molecules. 2022 Feb 14;27(4):1276. doi: 10.3390/molecules27041276 (PMC8879610; doi:10.3390/molecules27041276)
Supplement: Supplementary file 1 [file molecules-27-01276-s001.zip › molecules-1571316-supplementary.pdf]

molecules

Table S1. List of antibodies for western blotting (WB), immunohistochemistry (IHC), and immunocytochemistry (ICC).

Antigen (host)	Company	Catalog no.	Dilution rate
NRF2 (Rabbit)	Bioss	BS-1074R	1:300 (WB)<br>1:200 (IHC)
pNRF2 (Rabbit)	Bioss	BS-2013R	1:300 (WB)<br>1:200 (IHC)
HO-1 (Mouse)	Abcam	ab13248	1:250 (WB)<br>1:200 (IHC)
β-actin (Rabbit)	Cell signaling technology	4967s	1:500 (WB)
p53 (Mouse)	Oncogene	OP43	1:500 (WB)
MITF (Rabbit)	LSBio	LC-C117668	1:500 (WB)
NF-κB (Rabbit)	Cell signaling technology	8242s	1:400 (ICC)<br>1:400 (IHC)
COL1A1 (Mouse)	Santa cruz biotechnology	sc-293182	1:50 (IHC)
Fibrillin 1 (Mouse)	GeneTex	GTX23090	1:100 (IHC)
Fibrillin 2 (Mouse)	Santa cruz biotechnology	sc-393968	1:50 (IHC)
Fibulin 5 (Rabbit)	Proteintech	12188-1-AP	1:200 (IHC)

Table S2. List of primer for quantitative real time polymerase chain reaction. (qRT-PCR).

Gene		Primers
Actb	Forward	5'-CCGTAAAGACCTCTATGCCAAC-3′
	Reverse	5'-GCAGTAATCTCCTTCTGCATCC-3′
p53	Forward	5'-CAG ACT GAC TGC CTC TGC AT-3′
	Reverse	5'-CTT GGG CCA GGA ACC ACT AC-3′
Mitf	Forward	5'-GAG AAC TGC AGC CAG GAA CT-3′
	Reverse	5'-GCC GCA TTT AGA AAG CGA GA-3′
Mmp2	Forward	5'-GAT GAT GAC CGG AAG TGG GG-3′
	Reverse	5'-ATG AAG ATG ATA GGG CCC GTG-3′
Mmp3	Forward	5'-GGT GGC TTC AGT ACC TTC CC-3′
	Reverse	5'-GCC CTC GTA TAG CCC AGA AC-3′
Mmp9	Forward	5'-GGG TCT AGG CCC AGA GGT AA-3′
	Reverse	5'-TAA CGC CCA GTA GAG AGC CT-3′

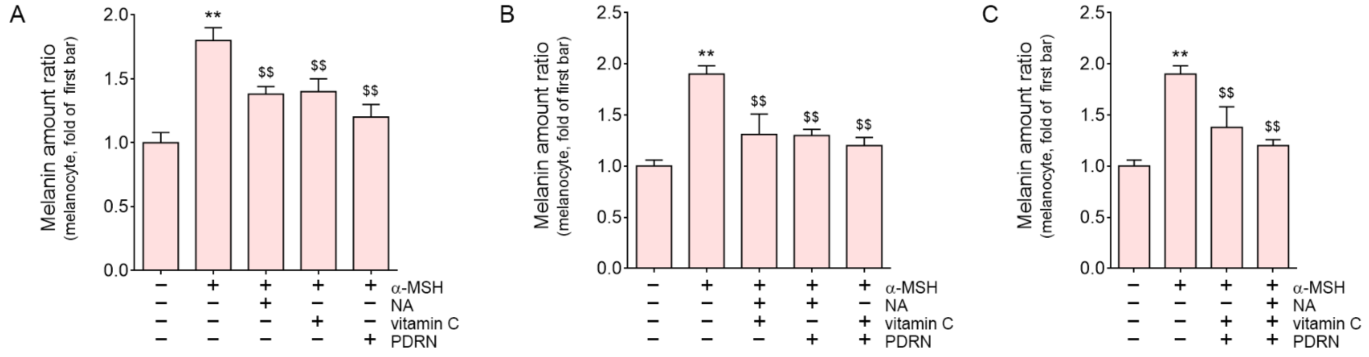

Figure S1. Regulation of melanin amount ratio by various combinations of niacinamide, vitamin C, and PDRN in the α-MSH- treated human melanocytes. (**A**) When single compound (niacinamide, vitamin C, or PDRN) were treated, the amount of melanin was determined in the α-MSH-treated human melanocytes. (**B**) Melanin amount ratio by combination of two compounds (vitamin C+niacinamide, PDRN+niacinamide, and vitamin C+PDRN) were confirmed in the α-MSH-treated human melanocytes. (**C**) Melanin amount ratio by vitamin C+PDRN and PVN were measured in the α-MSH-treated human melanocytes. Data are presented as the mean ± standard deviation. **, $p < 0.01$ vs. first bar; $$, $p < 0.01$ vs. second bar (Mann–Whitney U test). α-MSH, alpha-melanocyte stimulating hormone; NA, niacinamide; PDRN, polydeoxyribonucleotide, PVN, polydeoxyribonucleotide+vitamin C+niacinamide.

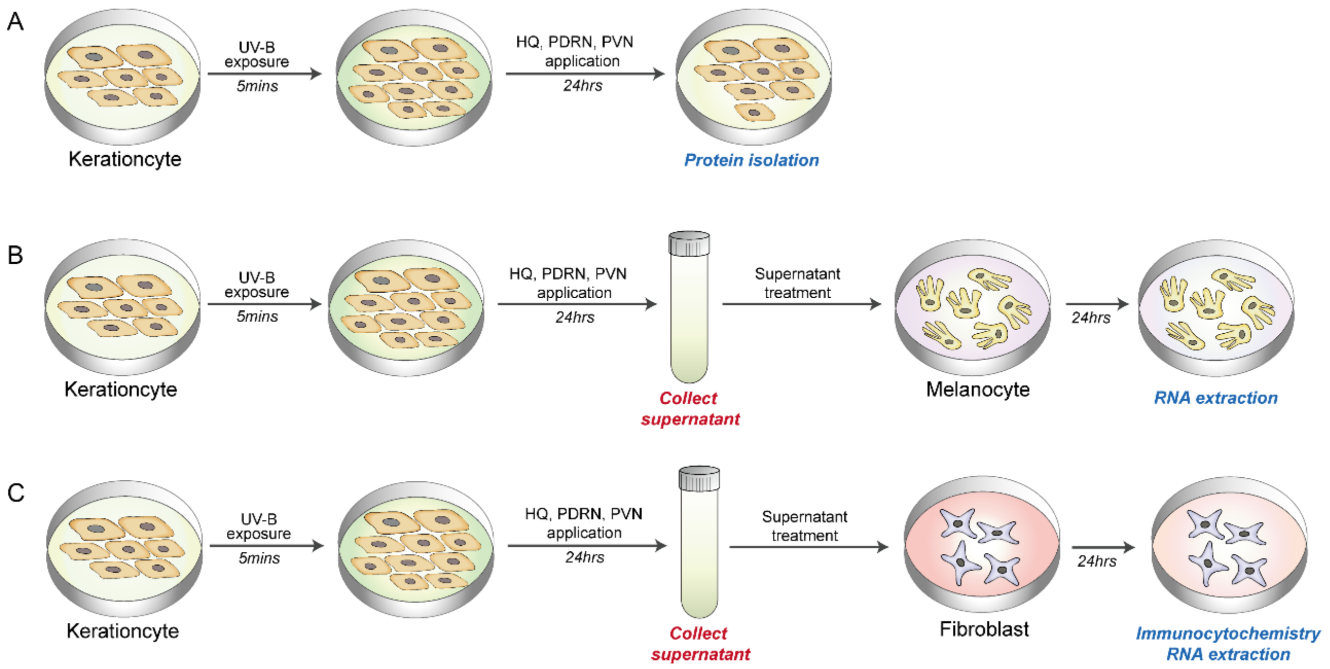

Figure S2. Schematic diagram of the in vitro experiment used in this study. (**A**) Human keratinocytes were exposed to UV-B (200mJ/cm^2) for 5 min and then HQ (100µM), PDRN (1mM), or PVN (1mM) was added to the UV-B-radiated keratinocytes for 24 h. (**B**) HQ, PDRN, or PVN was added to UV-B-radiated keratinocytes. After 24 h, the supernatant from those cell cultures was collected. CMs from keratinocytes or PBS were subsequently treated with melanocytes for 24 h, and then, RNA was extracted. (**C**) HQ, PDRN, and PVN were treated to UV-B-radiated keratinocytes and then collected the CM of the keratinocyte cultures was collected. CMs from keratinocytes were treated with fibroblasts for 24 h, immunocytochemistry was performed, and the mRNA level was evaluated. CM, conditioned medium; HQ, hydroquinone; PBS, phosphate-buffered saline; PDRN, polydeoxyribonucleotide; PVN, polydeoxyribonucleotide+vitamin C+niacinamide; UV-B, ultraviolet B.

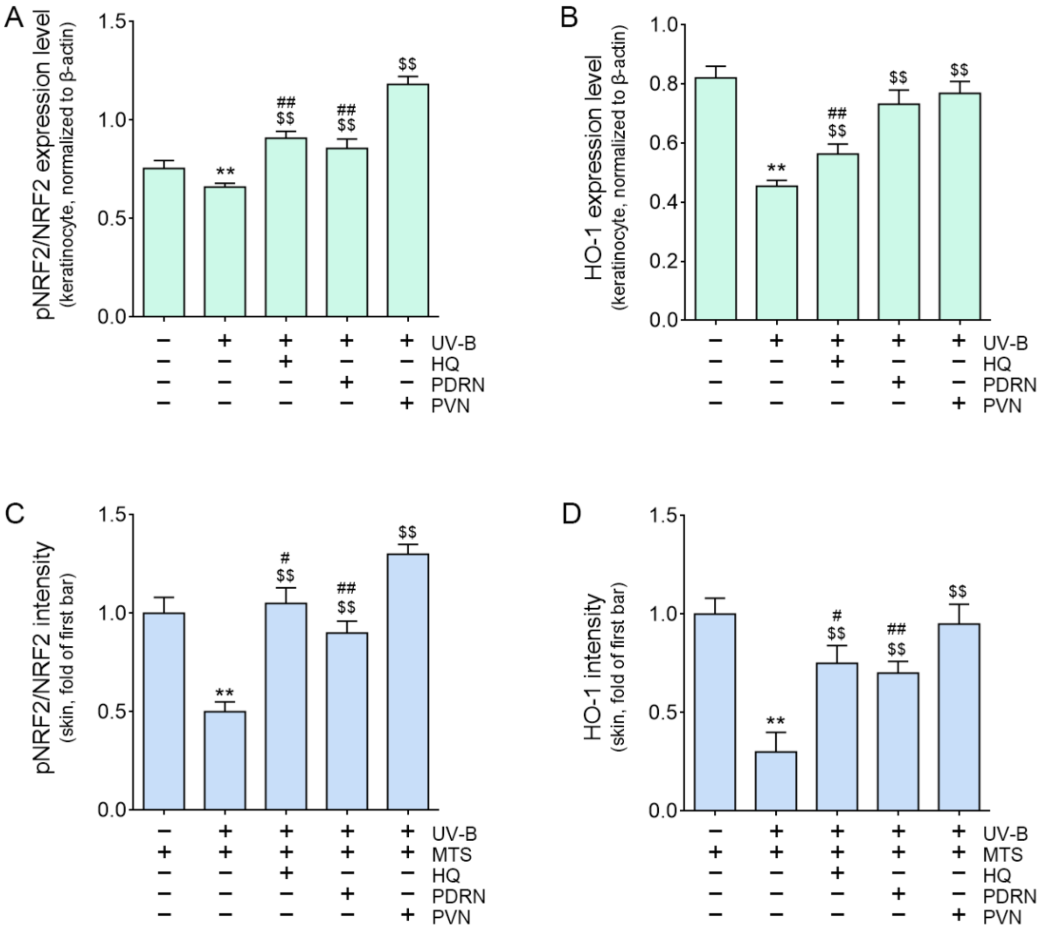

Figure S3. Regulation effect on the expression of NRF2 and HO-1 by HQ, PDRN, and PVN treatment in the UV-B-radiated human keratinocytes and animal skin. (**A** and **B**) The protein expression levels of pNRF2/NRF2 (**A**) and HO-1 (**B**) detected in the UV-B-radiated human keratinocytes were quantified by immunoblots – shown in Figure 1A. The band intensity was normalized to β-actin. (**C** and **D**) The protein expression levels of pNRF2/NRF2 (**C**) and HO-1 (**D**) developed in the epidermis of the UV-B-radiated animal skin were quantified by immunohistochemistry– shown in Figure 1B. The intensity was measured through brown staining by immunohistochemistry. Data are presented as the mean ± standard deviation. **, $p < 0.01$ vs. first bar; $$, $p < 0.01$ vs. second bar; #, $p < 0.05$ and ##, $p < 0.01$ vs. 5th bar (Mann–Whitney U test). HO-1, heme oxygenase-1; HQ, hydroquinone; MTS, microneedling treatment system; NRF2, nuclear factor erythroid-2-related factor 2; PDRN, polydeoxyribonucleotide; pNRF2, phosphorylated nuclear factor erythroid-2-related factor 2; PVN, polydeoxyribonucleotide+vitamin C+niacinamide; UV-B, ultraviolet B.

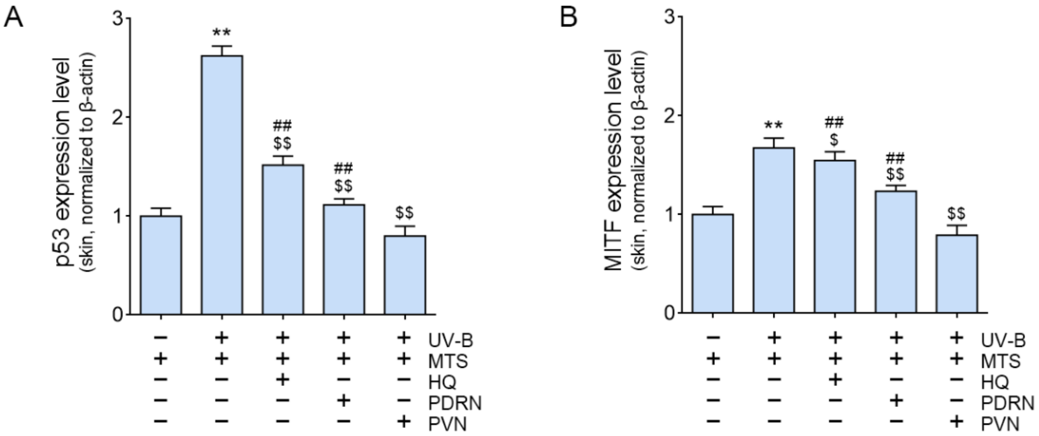

Figure S4. Regulation of the expression of p53 and MITF by HQ, PDRN, and PVN treatment in UV-B-radiated animal skin. (**A** and **B**) The protein expression levels of p53 (**A**) and MITF (**B**) were determined in the UV-B-radiated animal skin were quantified by immunoblots – shown in Figure 2E. The band intensity was normalized to β-actin. Data are presented as the mean ± standard deviation. **, $p < 0.01$ vs. first bar; $, $p < 0.05$ and $$, $p < 0.01$ vs. second bar; ##, $p < 0.01$ vs. 5th bar (Mann–Whitney U test). HQ, hydroquinone; MITF, microphthalmia-associated transcription factor; MTS, microneedling treatment system; PDRN, polydeoxyribonucleotide; PVN, polydeoxyribonucleotide+vitamin C+niacinamide; p53, tumor protein P53; UV-B, ultraviolet B.

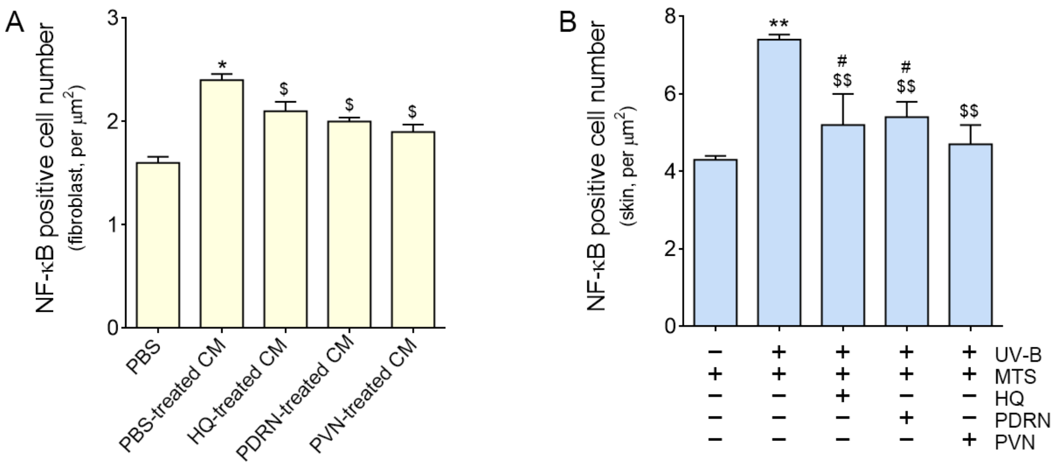

Figure S5. Regulation of the expression of NF-κB by HQ, PDRN, and PVN treatment in CM-treated human fibroblasts and animal skin. (**A**) The positive cell number of NF-κB was measured in the fibroblast treated with PBS or CM from UV-B-radiated keratinocytes. The number of NF-κB positive cells per μm^2 shown in Figure 3A was measured for the green signals in the nucleus by immunofluorescence. (**B**) The number of NF-κB positive cells in Figure 3E was counted as brown signals in the nucleus following immunohistochemistry staining. Data are presented as the mean ± standard deviation. *, $p < 0.05$ and **, $p < 0.01$ vs. first bar; $, $p < 0.05$ and $$, $p < 0.01$ vs. second bar; #, $p < 0.05$ vs. 5th bar (Mann–Whitney U test). CM, conditioned medium; HQ, hydroquinone; MTS, microneedling treatment system; NF-κB, nuclear factor kappa-light-chain-enhancer of activated B cells; PBS, phosphate-buffered saline; PDRN, polydeoxyribonucleotide; PVN, polydeoxyribonucleotide+vitamin C+niacinamide; UV-B, ultraviolet B.

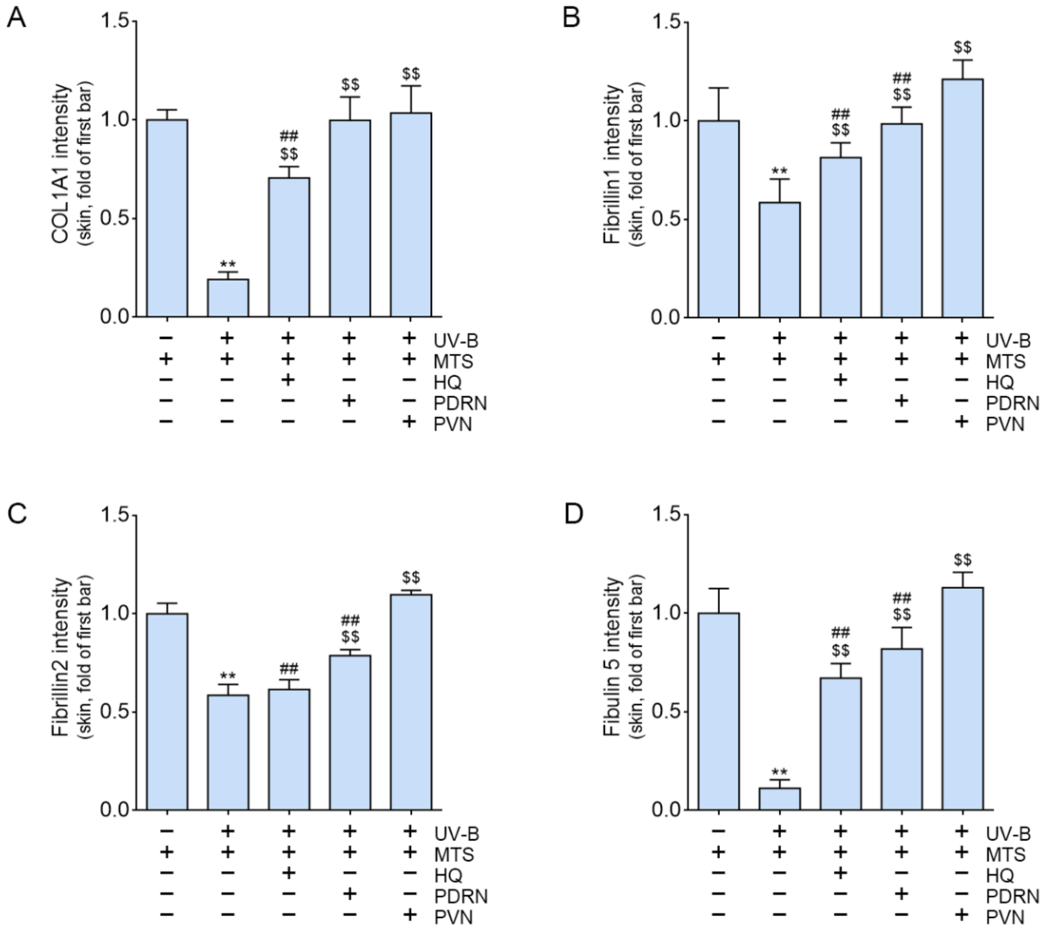

Figure S6. Regulation of the expression of COL1A1, Fibrillin 1/2, and Fibulin 5 by PVN treatment in UV-B-radiated animal skin. (**A**–**D**) The intensity of COL1A1, Fibrillin 1/2, and Fibulin 5 were determined in the UV-B-radiated animal skin by immunohistochemistry – shown in Figure 4A. The intensity was measured as brown signals. Data are presented as the mean ± standard deviation. **, $p < 0.01$ vs. first bar; $$, $p < 0.01$ vs. second bar; ##, $p < 0.01$ vs. 5th bar (Mann–Whitney U test). COL1A1, collagen Type I α1 chain; HQ, hydroquinone; MTS, microneedling treatment system; PDRN, polydeoxyribonucleotide; PVN, polydeoxyribonucleotide+vitamin C+niacinamide; UV-B, ultraviolet B.
